# Retinoic Acid Signaling Modulates Recipient Gut Barrier Integrity and Microbiota After Allogeneic Hematopoietic Stem Cell Transplantation in Mice

**DOI:** 10.3389/fimmu.2021.749002

**Published:** 2021-10-25

**Authors:** Pan Pan, Samantha N. Atkinson, Brian Taylor, Haojie Zhu, Dian Zhou, Philip Flejsierowicz, Li-Shu Wang, Matthew Morse, Chen Liu, Ian L. Gunsolus, Xiao Chen

**Affiliations:** ^1^ Division of Hematology & Oncology, Medical College of Wisconsin, Milwaukee, WI, United States; ^2^ Department of Medicine, Medical College of Wisconsin, Milwaukee, WI, United States; ^3^ Center for Microbiome Research, Medical College of Wisconsin, Milwaukee, WI, United States; ^4^ Department of Microbiology and Immunology, Medical College of Wisconsin, Milwaukee, WI, United States; ^5^ Department of Pathology, Yale University School of Medicine, New Haven, CT, United States; ^6^ Department of Pathology, Medical College of Wisconsin, Milwaukee, WI, United States

**Keywords:** retinoic acid, intestinal barrier, gut microbiota, vitamin A, graft-versus-host disease

## Abstract

Graft-versus-host disease (GVHD) remains a major complication after allogeneic hematopoietic stem cell transplantation (HSCT). An impaired intestinal epithelial barrier is an important component of GVHD pathogenesis. However, contributing host factors that modulate mucosal barrier integrity during GVHD are poorly defined. We hypothesized that vitamin A and retinoic acid (RA) exert positive impacts on maintaining intestinal barrier function after HSCT, thus preventing or dampening GVHD severity. Unexpectedly, we found that exogenous RA increased intestinal permeability of recipient mice after allogeneic HSCT. Serum bacterial endotoxin levels were significantly higher in GVHD mice fed a vitamin A-high (VAH) diet compared to those fed a vitamin A-normal (VAN) diet, indicating a more compromised intestinal barrier function. Furthermore, VAH mice showed more severe lung GVHD with increased donor T cell infiltration in this tissue and died significantly faster than VAN recipients. 16S rRNA sequencing of fecal samples revealed significant differences in the diversity and composition of gut microbiota between VAN and VAH transplant recipients. Collectively, we show that retinoic acid signaling may negatively impact intestinal barrier function during GVHD. Mild vitamin A supplementation is associated with increased lung GVHD and more profound gut dysbiosis. Micronutrients such as vitamin A could modulate complications of allogeneic HSCT, which may be mediated by shaping gut microbiota.

## Introduction

Graft-*versus*-host disease (GVHD) remains a major complication after allogeneic hematopoietic stem cell transplantation (HSCT) (1, 2). The gastrointestinal (GI) tract is one of the major target organs of GVHD and its involvement is often associated with a poor prognosis (3–5). Since the GI tract is home to an enormous amount of microbiota, its damage from the pretransplant conditioning regimen and subsequent alloimmunity can lead to leakage of immunostimulatory gram-negative bacterial fragments, such as lipopolysaccharide (LPS), into the circulation that can intensify the inflammatory responses characteristic of GVHD (6). In this regard, it has been shown that a breached mucosal epithelial barrier is an important component of GVHD pathogenesis (7, 8). Accumulating evidence also shows that protecting or restoring intestinal epithelial barrier function may be an effective approach in preventing or mitigating GVHD (9–11). Such a strategy is appealing because it deviates from conventional GVHD management by targeting recipient nonimmune cells instead of donor immune cells, such as T cells and antigen presenting cells (12–14). Since this strategy does not interfere with donor T cell activation and function, one can expect maximal graft-versus-leukemia (GVL) response without overt GVHD. However, host factors that are involved in maintaining and modulating intestinal barrier function after allogeneic HSCT are not well defined.

Vitamin A is an essential nutrient that participates in a variety of biological processes (15). In particular, it is well established that vitamin A is an important factor that helps maintain mucosal epithelial barrier function. An epithelial monolayer of cells and tight junctions are two major components that form the intestinal mucosal barrier. Tight junctions are important proteins that seal adjacent epithelial cells thus preventing the leakage of solutes and water through paracellular pathways. Vitamin A has been shown to upregulate *ZO-1, Occludin, and Claudin* tight junction mRNA and protein expression (16). Vitamin A deficiency increases the risk of GI tract infection and vitamin A supplementation in children can significantly reduce mortality associated with diarrhea (17). In the context of HSCT, we and others have shown that retinoic acid (RA), the active metabolite of vitamin A, actively participates in regulating GVHD severity (18, 19). In these preclinical studies, the RA signaling in donor T cells modulates intestinal GVHD risk by influencing donor T cell polarization and migration. Exogenous RA intensifies GVHD and leads to increased mortality in mice undergoing allogeneic HSCT (18–20). In addition, it has been shown that host dendritic cell RA signaling regulates GVHD severity (21, 22). However, whether RA signaling affects intestinal barrier function after allogeneic HSCT remains unclear. This is a clinically relevant question since recipient vitamin A levels could potentially be modified to strengthen the intestinal barrier and mitigate GVHD.

We hypothesized that vitamin A and RA exert positive impacts on maintaining intestinal epithelial integrity during GVHD. In this study, we tested this hypothesis with dietary modification of vitamin A levels in recipient mice and examined how a clinically relevant dose of vitamin A supplementation affects GVHD risk. Contrary to our expectation, exogenous RA and mild vitamin A supplementation increased intestinal permeability after allogeneic HSCT. Vitamin A supplementation was associated with more severe lung damage and increased mortality of recipient mice after transplantation. Fecal bacterial analysis revealed significant changes in diversity and composition of microbiota in these animals. These results demonstrate that RA signaling has the potential of modifying recipient intestinal barrier integrity and gut microbiota after allogeneic HSCT. Furthermore, a diet with higher than normal vitamin A levels was associated with worse clinical outcomes in our GVHD model. These results have important implications for the potential use of high-dose vitamin A in the clinical setting of GVHD.

## Materials and Methods

### Mice

C57BL/6 (B6; H-2K^b^) and Balb/c (H-2K^d^) mice were purchased from The Jackson Laboratory (Bar Harbor, ME) or bred in the Biomedical Resource Center at the Medical College of Wisconsin (MCW). All experiments and procedures were carried out under protocols approved by the MCW Institutional Animal Care and Use Committee.

### Animal Experiments

Bone marrow transplantation (BMT) was performed as described previously (23). Briefly, bone marrow (BM) was flushed from femurs and tibias of B6 donor mice with Dulbecco′s Modified Eagle′s Medium (DMEM) and passed through sterile mesh filters to obtain single-cell suspensions. Splenic Pan-T cells were purified from donor mice using EasySep™ Mouse T Cell Isolation Kit (StemCell Technologies, Cambridge, MA) according to the manufacturer’s instructions. Balb/c recipients were conditioned with total body irradiation administered as a single exposure of 900 Rads using a Shepherd Mark I Cesium Irradiator (J. L. Shepherd and Associates, San Fernando, CA). Irradiated recipients received a single intravenous injection of bone marrow cells (6–10 ×10^6^) plus purified splenic Pan-T cells (0.3–0.5 ×10^6^) in the lateral tail vein. Exogenous RA was given to recipient mice *via* intraperitoneal injection after BMT. For experimental purpose of vitamin A dietary modification, 3-week old male Balb/c mice were randomized into two groups and were fed a AIN-93G-based diet containing either normal (4 IU/g, VAN) or higher (10 IU/g, VAH) amount of vitamin A (Envigo, Indianapolis, IN) for at least additional 8 weeks. BMT was performed on these recipient mice as described above.

### Isolation of Colonic Epithelium

Colon tissues were harvested upon termination of studies. Colonic epithelium was isolated according to a modified protocol (24). Briefly, the dissected colons were cut longitudinally, washed in cold PBS, and incubated in ice-cold BD Cell Recovery Solution (BD Biosciences) for 10 minutes. Colon tissues were then transferred to 2 ml of cold PBS and gently peeled/scraped by fine needles or scalpels. Detached epithelial layers were collected by centrifugation at 1600 rpm for 5 minutes.

### Caco-2 Cultures and Experiments

Caco-2 cells (HTB-37™, Lot# 70013347) were purchased from the American Type Culture Collection (ATCC, Manassas, VA). Caco-2 cells were grown in DMEM supplemented with 10% heat-inactivated FBS, 2 mM L-glutamine, 1% Non-Essential Amino Acids (NEAA), and 1% penicillin/streptomycin in a humidified 37°C, 5% CO_2_ incubator. The culture medium was changed every two to three days. Caco-2 cells were grown in 100-mm culture dishes (Thermo Fisher Scientific, Waltham, MA) and sub-cultured by digestion with 0.25% trypsin-EDTA. For experimental purpose, Caco-2 cells were cultured and maintained in 24-well plates for 14–21 days to differentiate before treatment. Cells were treated with TNF-α (20 ng/mL) and IL-6 (20 ng/mL) for 72 hours in the presence or absence of 5 µM RA. Untreated cells were used as controls. Cultures were collected for RNA isolation, reverse transcription, and quantitative real-time PCR analysis.

### Quantitative Real-Time PCR Analysis

The TRIZOL^®^ reagent (Sigma-Aldrich) was used to homogenize tissues/cells and isolate total RNA according to the manufacturer’s instructions. The concentration of total RNA was determined by absorbance at 260/280 nm using NanoDrop. Reverse transcription was performed using QuantiTect^®^ Reverse Transcription Kit (Qiagen, Hilden, Germany). Relative gene expression was measured using QuantiTect^®^ SYBR Green PCR Kit (Qiagen). β-2-microglobulin (B2M) and glyceraldehyde-3-phosphate dehydrogenase (GAPDH) were used as the housekeeping genes for mouse studies and Caco-2 studies, respectively.

### Flow Cytometric Analysis

Immune cells were labeled with monoclonal antibodies conjugated to fluorescein isothiocyanate (FITC), phycoerythrin (PE), PE-Cy5, PE-Cy7, or allophycocyanin (APC). PE-anti-CD4 (clone GK1.5), FITC-anti-CD8a (clone 53-6.7), PE-anti-TCRβ (clone H57-597), FITC-anti-H-2K^b^ (clone AF6-88.5), APC-anti-CD8a (clone 53-6.7), and PE-Cy5-anti-CD8a (clone 53-6.7) were purchased from BD Biosciences. FITC-anti-CCR9 (clone CW-1.2), PE-anti-α4β7 (clone DATK32), PE-Cy7-anti-CD4 (clone GK1.5), and APC-anti-H-2K^b^ (clone AF6-88.5) were obtained from eBioscience (San Diego, CA). Samples were analyzed using a BD LSRII flow cytometer. Data were analyzed using FlowJo software (TreeStar, Ashland, OR).

### Cytometric Bead Array

Serum cytokine levels were determined using the BD Cytometric Bead Array system (BD Biosciences) according to the manufacturer’s protocol.

### Serum FITC-Dextran Analysis

After food and water were withheld for 3-4 hours, mice were orally administered with 0.75 mg/g body weight of 4kDa FITC-dextran (Sigma-Aldrich). Serum samples were collected 3-4 hours after oral administration. Fluorescence was measured spectrophotometrically in 96-well opaque plates (excitation: 485 nm, emission: 528 nm). Serum FITC-dextran levels were determined based on generated standard curves.

### Serum LPS Analysis

Serum LPS levels were measured using Pierce™ LAL Chromogenic Endotoxin Quantitation Kit (Thermo Fisher Scientific) according to the manufacturer’s instructions.

### Pathological Analysis

Representative samples of liver, colon, small intestine, and lung were fixed in formalin and paraffin embedded. The tissue sections were then stained with hematoxylin and eosin. Histological analysis was performed by an experienced pathologist (C.L.) in a blinded fashion. A semiquantitative scoring system was used to account for histological changes in the liver, colon, small intestine, and lung as previously described (25).

### Gut Microbiome Analysis

Fecal samples were collected immediately before termination of studies and genomic DNA was purified using DNeasy PowerLyzer PowerSoil Kit (Qiagen) according to the manufacturer’s instructions. Purified genomic DNA was sent to the University of Wisconsin-Madison Biotechnology Center and the V3/V4 variable region of the 16S rDNA gene was sequenced with 2x300bp paired end technology. The site-specific primers used were: 341F:5’-CCTACGGGNGGCWGCAG-3’ and 806R:5’-GACTACHVGGGTATCTAATCC-3’. Bioinformatic analyses of microbiome diversity, abundance, and functional pathways were performed by a senior Bioinformatics Analyst (S.N.A.) in a blinded fashion at the Center for Microbiome Research at the MCW. QIIME2 (v. 2020.2) was used to analyze the paired-end 16S rRNA sequencing reads (26). Sequences were imported, trimmed using CutAdapt, and summarized to check quality. Representative sequences were chosen using DADA2, which also removes chimeric sequences. The representative sequences were then aligned, masked for hypervariable regions, and phylogenetic trees were produced. A classifier was generated to assign taxonomy to the reads using the 99% similarity files of the SILVA 132 release and the 341-806 region of the 16S gene. Taxonomy was assigned to the feature table to make taxonomy bar plots and to generate relative abundance tables. Diversity metrics were run using the *core-metrics-phylogenetic* command of QIIME2. Alpha and beta diversity were analyzed using their respective commands, alpha/beta-group-significance (27). Alpha and beta diversity boxplots were generated using R. Principal Coordinate Analysis (PCoA) plots were examined using Emperor (28) and finalized figures were made using the qiime2R package in R. LEfSe, Linear Discriminant Analysis (LDA) effect size, was run to determine enriched organisms from each group (29). PICRUSt2 was used to predict functional capacity of the 16S reads (30, 31). Output from PICRUSt2 was then put through LEfSe to determine differentially abundant functional predictions.

### Hepatic Function Assay

Serum samples were collected upon termination of studies and sent to Wisconsin Diagnostic Laboratories for hepatic function assay. Levels of alanine aminotransferase (ALT) and aspartate aminotransferase (AST) were determined using Roche cobas c702 analyzers.

### Statistical Analysis

Statistical analysis was performed using GraphPad Prism (La Jolla, CA). Survival comparisons were performed using the log-rank test. Other differences between experimental groups were analyzed using an unpaired two-tailed Student’s *t*-test or one-way ANOVA. Mann-Whitney *U-*test was used for group comparison in microbiota experiments. A *p* value less than 0.05 was considered as statistical significance in all experiments.

## Results

### Retinoic Acid Increases Gut Permeability of Recipient Mice After Allogeneic BMT

**Figure 1:**
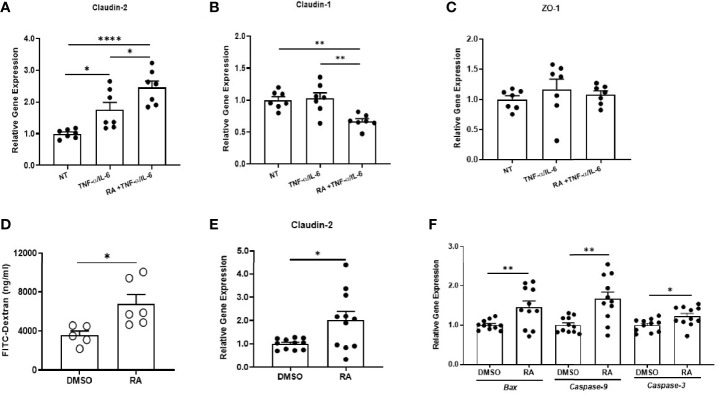
Retinoic acid increases gut permeability of recipient mice after allogeneic BMT. Caco-2 cells were maintained for 14–21 days and allowed to differentiate. The cultures were then treated with either DMSO or 5 µM RA for 4 days. They were exposed to TNF-α (20 ng/ml) and IL-6 (20 ng/ml) during the last 24 hours of incubation. Culture wells that did not receive any treatments were used as controls. Gene expression of *Claudin-2* **(A)**, *Claudin-1* **(B)**, *and ZO-1* **(C)** from different treatment groups was analyzed by real-time q-PCR. Data are normalized for β_2_‐microglobulin RNA and presented as fold increase over gene expression in no treatment (NT) group. Data are shown as Mean ± SEM and are cumulative results from two independent experiments. **(D–F)** Lethally irradiated Balb/c mice were transplanted with 7 x10^6^ BM plus 0.4 x 10^6^ purified T cells from B6 mice. Recipient mice then received intraperitoneal injections of either DMSO or 450 µg of RA every other day for 4 doses. On Day 7 post-BMT, FITC-dextran analysis was performed, and colonic epithelium was collected for real-time q-PCR analysis. **(D)** Serum levels of FITC-dextran in recipient mice (n = 5–6 per group). **(E, F)** Relative gene expression of *Claudin-2* and apoptotic markers (n = 11 per group). Data are shown as Mean ± SEM and are cumulative results from two to three independent experiments. Statistics: *P ≤.05, **P ≤.01, ****P ≤.0001.

We first examined the effects of RA, the active metabolite of vitamin A, on intestinal barrier function in a culture system. To that end, we used Caco-2 cells, a widely used human intestinal epithelial cell line. TNF-α and IL-6, two cytokines that are elevated in many gastrointestinal inflammatory disorders including GVHD, have been shown to increase the permeability of Caco-2 cells (32, 33). Consistent with these findings, treating Caco-2 cells with TNF-α and IL-6 led to a significant increase in gene expression of *Claudin-2*, a tight junction molecule that is associated with increased intestinal permeability (34). It has been well documented that Claudin-2 is a pore-forming protein that contributes to inducing a “leaky gut” in several intestinal inflammatory disorders (35). Interestingly, the expression of *Claudin-2* was further increased in the presence of RA (**Figure 1A**). We also measured gene expression of other tight junction molecules including *Claudin-1 and ZO-1*. There was a significant reduction in gene expression of *Claudin-1* in Caco-2 cells after RA exposure, whereas *ZO-1* expression levels were not affected (**Figures 1B, C**). These results suggest that RA may further impair gut barrier integrity in an inflammatory environment by increasing Claudin-2 expression.

We then investigated the effect of RA on intestinal barrier function in recipient mice undergoing allogeneic BMT. We treated recipient mice with RA or DMSO after transplantation and measured intestinal permeability by fluorescein isothiocyanate (FITC)-dextran assay. In this assay, the translocation of orally applied FITC-dextran into circulation is measured to reflect intestinal barrier integrity. On day 7 after transplantation, serum FITC-dextran levels were significantly higher in RA-treated mice compared to DMSO-treated mice (**Figure 1D**), demonstrating impaired gut barrier integrity. We further isolated the colonic epithelium and measured gene expression levels of tight junction molecules. Consistent with *in vitro* studies, RA treatment significantly increased the expression of *Claudin-2* in purified colon epithelial cells (**Figure 1E**). In addition, gene expression of pro-apoptotic marker *Bax* and caspase family members (*Caspase-3* and *Caspase-9*) was significantly increased in RA-treated mice (**Figure 1F**). These results indicate that exogenous RA can negatively impact intestinal barrier function of allogeneic BMT recipients by increasing Claudin-2 expression and promoting the apoptosis of intestinal epithelial cells.

### Recipient Mice Fed a Diet High in Vitamin A Show Increased Gut Permeability After Allogenic BMT

**Figure 2:**
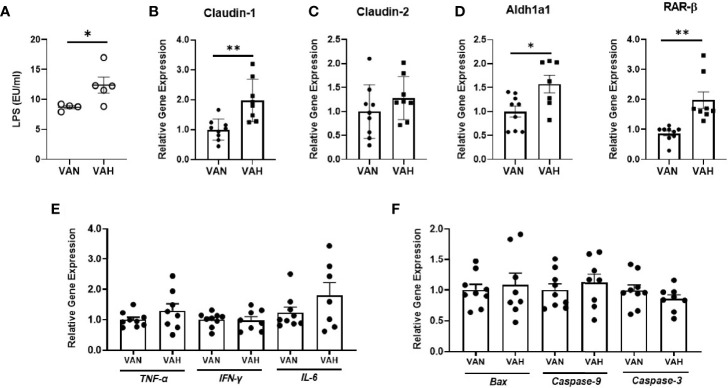
Recipient mice fed a diet high in vitamin A show increased gut permeability after allogenic BMT. Lethally irradiated VAN and VAH mice were transplanted with BM (6–10 x 10^6^) plus 0.3–0.4 x 10^6^ purified T cells from B6 mice. On Day 7 post-BMT, serum and colonic epithelium were collected. **(A)** Serum LPS levels in recipient mice (n = 4–5 per group). Data are shown as Mean ± SEM and are derived from one of two representative experiments. **(B–F)** Relative gene expression of *Claudin-1*, *Claudin-2, aldh1a1, RAR-β*, inflammatory cytokines, and apoptotic markers of colonic epithelial cells (n = 8–9 per group). Data are shown as Mean ± SEM and are cumulative results from three independent experiments. Statistics: *P ≤.05, **P ≤.01.

To examine how endogenous RA signaling modulates intestinal barrier function after allogeneic BMT, we generated vitamin A normal (VAN) and vitamin A high (VAH) Balb/c recipient mice through dietary modifications. In these studies, VAH mouse chow contained 2.5-fold higher vitamin A levels compared to VAN mouse chow. Pretransplant conditioning and subsequent alloimmunity can cause significant damage to host intestinal epithelial barrier, leading to translocation of LPS into the systemic circulation. Thus, serum LPS levels reflect the integrity of intestinal epithelial barrier. On day 7 after transplantation, serum LPS levels were significantly higher in VAH recipients compared to those of VAN mice (**Figure 2A**), indicating a more compromised intestinal barrier. Gene expression of tight junction molecule *Claudin-1* was also significantly higher in VAH mice compared to that of VAN mice (**Figure 2B**). However, there was no significant difference in *Claudin-2* expression in intestinal epithelial cells on day 7 after BMT (**Figure 2C**). Interestingly, mRNA levels of genes associated with RA biosynthesis and signaling, such as *aldh1a1* and *RAR-β*, were significantly higher in colon epithelial cells of VAH mice (**Figure 2D**). Examination of inflammatory cytokines and apoptosis-associated markers also did not reveal differences between the two groups (**Figures 2E, F**). Thus, vitamin A supplementation is associatedwith decreased intestinal barrier integrity without affectingClaudin-2 expression.

### Dietary Vitamin A Supplementation Does Not Affect Donor T-Cell Compartment During GVHD

**Figure 3:**
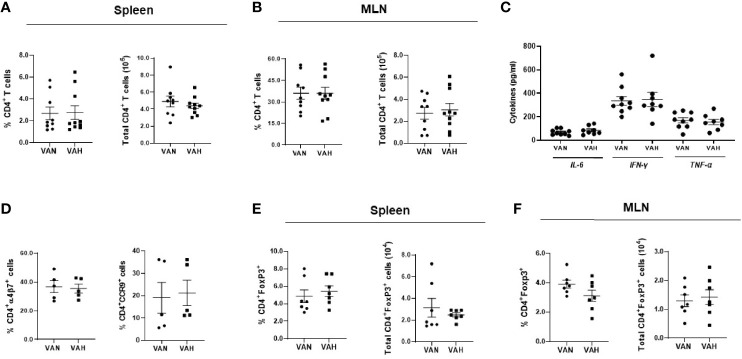
Dietary vitamin A supplementation does not affect donor T-cell compartment after allogeneic BMT. Lethally irradiated VAN and VAH mice were transplanted with BM (6–10 x 10^6^) plus 0.3–0.4 x 10^6^ purified T cells from B6 mice. On Day 7 post-BMT, the percentage and absolute number of donor H‐2^b+^CD4^+^ T cells in the spleen **(A)** and MLNs **(B)** were examined. **(C)** Serum proinflammatory cytokine levels were determined by cytometric bead array. Data are shown as Mean ± SEM and are cumulative results from three independent experiments. **(D)** The percentage of donor H‐2^b+^CD4^+^ T cells expressing α4β7 and CCR9 from MLNs of recipient mice was examined. Data are shown as Mean ± SEM and are cumulative results from two experiments. **(E, F)** BMT was performed as described above except Foxp3^+^EGFP^+^ mice were used as donors. On Day 7 post-BMT, the percentage and absolute number of donor H‐2^b+^CD4^+^Foxp3^+^ Tregs in the spleen **(E)** and MLNs **(F)** were examined (n = 7 per group). Data are shown as Mean ± SEM and are cumulative results from two independent experiments.

To determine how vitamin A supplementation influences the *in vivo* expansion of donor T cells, we harvested the spleen and mesenteric lymph nodes (MLNs) of VAN and VAH mice on day 7 after BMT. There were no significant differences in the percentage and absolute number of donor CD4 and CD8 T cells in the spleen and MLNs between VAN and VAH mice (**Figures 3A, B**, and data not shown). There were also no significant differences in serum inflammatory cytokine levels between the two groups (**Figure 3C**). Retinoic acid is known to imprint gut-homing specificity on T cells by increasing the expression of integrin α4β7 and chemokine receptor CCR9. However, the percentage and absolute number of donor CD4 and CD8 T cells expressing these gut-homing molecules were similar between the two groups of mice (**Figure 3D** and data not shown). These results indicate that vitamin A supplementation does not appear to affect the expansion and gut-homing potential of donor T cells at the early stages of GVHD development. Since RA plays an important role in facilitating the induction of Foxp3^+^ regulatory T cells (Tregs), we determined how vitamin A supplementation affects donor Treg reconstitution after transplantation. Using purified CD4^+^ T cells from Foxp3^+^EGFP^+^ donor mice, we found no significant differences in the percentage and absolute number of CD4^+^Foxp3^+^EGFP^+^ Tregs in the spleen and MLNs between VAN and VAH recipients (**Figures 3E, F**). Collectively, these data indicate that vitamin A supplementation does not affect the expansion of donor T cells and the reconstitution of Tregs after allogeneic BMT.

### Recipient Mice Fed a Diet High in Vitamin A Show More Severe Lung GVHD After Allogeneic BMT

**Figure 4:**
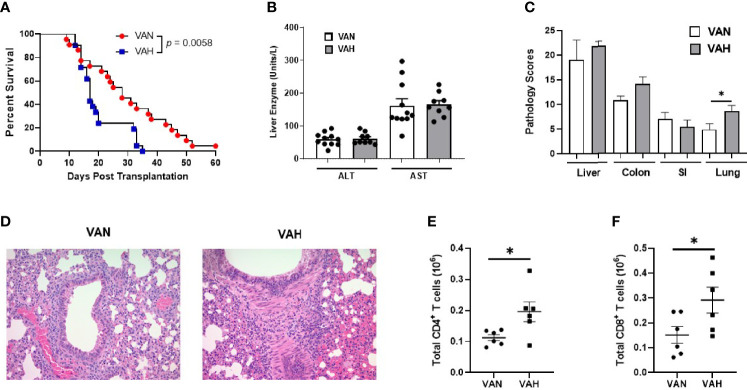
Recipient mice fed a diet high in vitamin A show more severe lung GVHD after allogeneic BMT. **(A)** Lethally irradiated VAN and VAH mice were transplanted with BM (7–8 x 10^6^) plus 0.4 x 10^6^ purified T cells from B6 mice. Overall survival is depicted. Data are the cumulative results from 7 independent experiments (n = 21–22 per group). **(B)** Serum liver enzymes in VAN and VAH recipients 7 days after transplantation. Data are shown as Mean ± SEM and are cumulative results from three independent experiments (n = 9–11 per group). **(C)** Recipient mice were euthanized on day 14 after BMT and pathologic damage in the liver, lung, small intestine, and colon was examined. Data are derived from one representative experiment (n = 5 per group). **(D)** Representative photo micrographs of the lungs from VAN and VAH mice are shown. **(E, F)** The absolute numbers of donor H‐2^b+^CD4^+^ T cells **(E)** and donor H‐2^b+^CD8^+^ T cells **(F)** in the lungs of recipient mice were examined. Data are shown as Mean ± SEM and are cumulative results from two experiments (n = 6 per group). Statistics: *P ≤ .05.

We then examined how vitamin A supplementation affects overall GVHD risk. We found that VAH BMT recipients showed increased mortality after transplantation and died significantly faster than VAN mice (**Figure 4A**). Since previous studies have indicated a potential role of vitamin A in regulating liver damage during GVHD (36), we measured liver enzymes alanine aminotransferase (ALT) and aspartate aminotransferase (AST) levels in recipient mice. There were no significant differences in serum ALT and AST levels between VAN and VAH mice (**Figure 4B**), indicating that early mortality of VAH mice is not attributable to increased hepatic GVHD. Pathological analysis of GVHD target organs on day 14 after transplantation revealed a significant increase in inflammatory changes of the lungs of VAH mice compared with VAN mice (**Figure 4C**), whereas there were no significant differences in pathology scores of the liver, colon, and small intestine between the two groups (**Figure 4C**). Specifically, there was lymphocytic infiltration around pulmonary vessels and bronchioles as well as pneumonitis, characteristic pathological features of GVHD, in both groups of mice. However, the extent and severity of these abnormalities were significantly increased in the lungs of VAH mice compared with VAN mice (**Figure 4D**). We further analyzed the immune cells infiltrating the lungs of VAN and VAH mice on day 7 after transplantation. We found significantly higher absolute numbers of donor CD4 and CD8 T cells in the lungs of VAH mice versus VAN mice (**Figures 4E, F**). Thus, mild vitamin A supplementation is associated with more severe lung GVHD with increased donor T cell infiltration in this tissue site.

### Dietary Vitamin A Supplementation Modulates Recipient Gut Microbiota After Allogeneic BMT

**Figure 5:**
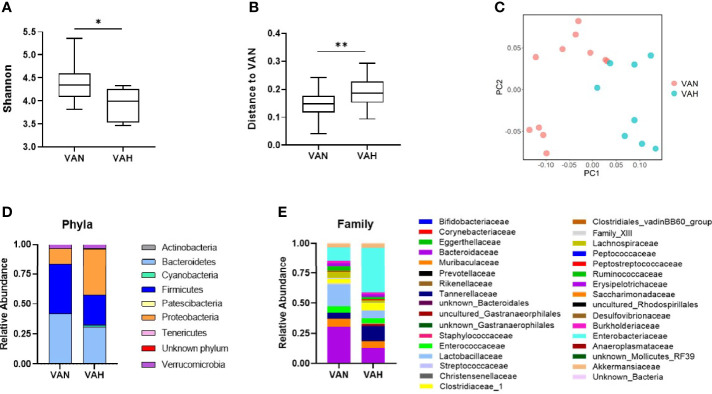
Dietary vitamin A supplementation modulates recipient gut microbiota after allogeneic BMT. **(A)** Lethally irradiated VAN and VAH mice were transplanted with BM (7 x 10^6^) plus 0.3–0.4 x 10^6^ purified T cells from B6 mice. On Day 7 after BMT, fecal samples were collected and 16S rRNA sequencing was performed. **(A)** Shannon index of gut microbiota as a measure of alpha diversity; **(B)** Weighted UniFrac analysis of microbiota β-diversity. (n = 9–11 per group). Data are shown as Mean ± SEM and are cumulative results from 3 independent experiments. **(C)** Principle coordinate analysis (PCoA) using Weighted UniFrac distance of gut microbiota sourced from VAN and VAH mice reveal beta diversity differences; **(D)** Relative abundance of microbial community at the phylum level. **(E)** Relative abundance of microbial community at the family level. Statistics: *P ≤.05, **P ≤.01.

**Figure 6:**
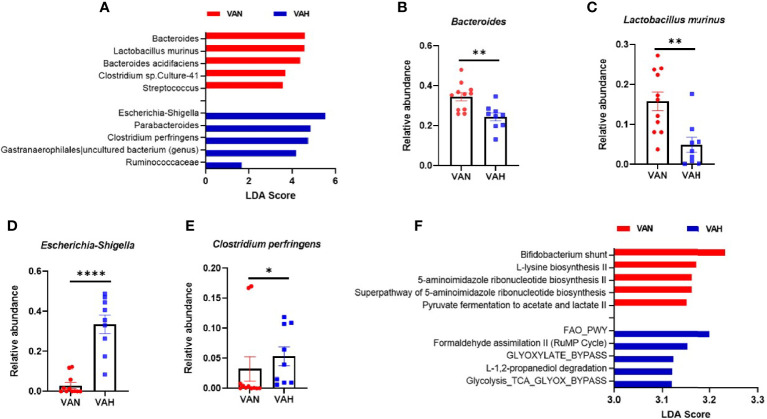
Vitamin A supplementation causes more profound gut dysbiosis with functional consequences in BMT recipients. **(A)** Lethally irradiated VAN and VAH mice were transplanted with BM (6–10 x 10^6^) plus 0.3–0.4 x 10^6^ purified T cells from B6 mice. On Day 7 after BMT, fecal samples were collected and 16S rRNA sequencing was performed. **(A)** Taxonomic comparison at the genus level using linear discriminant analysis effect size analysis. **(B–E)** Comparison of relative abundance of taxa at the genus level between the two groups. **(B)** *Bacteroides;* **(C)** *Lactobacillus murinus*; **(D)** *Escherichia-Shigella*; **(E)** *Clostridium perfringens*; (n = 9–11 per group). Data are shown as Mean ± SEM and are cumulative results from 3 independent experiments. **(F)** Linear discriminant analysis effect size analysis for the predicted metabolic pathways (KEGG modules) from two groups. Top 5 pathways with an LDA score greater than 3 from each group are shown. Statistics: *P ≤.05, **P ≤.01, ****P ≤.0001.

Diet is one of the most important factors that shape gut microbiota. We performed 16S ribosomal RNA gene sequencing of fecal samples from recipient mice 7 days after transplantation. We found that fecal samples from VAH mice had significantly lower alpha diversity compared to VAN recipient mice as shown by Shannon index (**Figures 5A**). In addition, there was a significant difference in beta diversity between the two groups (**Figure 5B**). Principal coordinates analysis (PCoA) based on weighted UniFrac distance revealed significantly different microbiota communities between VAN and VAH recipient mice. The samples from VAH group clustered separately from those of VAN mice (**Figure 5C**). These results indicated that vitamin A supplementation is associated with more severe loss of gut microbiota diversity, a hallmark of GVHD. There were also noticeable differences in relative abundance of fecal bacteria between the two groups. At the phyla level, VAH mice had increased abundance of the *Proteobacteria* phylum and decreased abundance of the *Firmicutes* phylum (**Figure 5D**). At the family level, *Lactobacillaceae* and *Bacteroidaceae* were decreased and *Enterobacteriaceae* was increased in VAH group (**Figure 5E**). At the genus level, there was specific enrichment of microbiota of VAN mice that are commonly associated with a healthier gut microenvironment. For example*, Bacteroides* and *Lactobacillus murinus* were the top two enriched genera that were significantly increased in these animals (**Figures 6A–C**). In contrast, there was an expansion of opportunistic pathogens including *Escherichia-Shigella* and *Clostridium perfringens* in VAH mice (**Figures 6A, D, E**). Finally, functional pathway analysis by PICRUSt revealed distinct metabolic pathways between the VAN and VAH groups. Specifically, carbohydrate metabolism with *Bifidobacteria* and metabolism of certain amino acids were increased in VAN mice, whereas pathways associated with fatty acid oxidation and formaldehyde assimilation were increased in VAH mice (**Figure 6F**). Thus, vitamin A supplementation is associated with compositional and functional alterations of gut microbiota after transplantation.

## Discussion

There is growing interest in mitigating GVHD through protecting or restoring the gut mucosal barrier that is damaged after allogeneic HSCT (7–9). Such an approach is expected to lead to a maximal GVL response with significantly diminished GVHD. It is conceivable that certain host factors may contribute to modulating the integrity of intestinal barrier in patients undergoing allogeneic HSCT. Research efforts to identify these host factors with the hope of targeting them to reduce GVHD are of significant clinical relevance. We are interested in how micronutrients, in particular vitamin A, regulates this process given the well-established beneficial effects of this molecule in maintaining mucosal barrier function. Indeed, recent studies have shown the crucial role of intestinal epithelial retinoid signaling in regulating the survival of mice in the context of GI infection (37). In this paper, we provide novel insights into how retinoic acid signaling influences intestinal tight junctions and microbiota in the setting of allogeneic HSCT.

The intestinal epithelial barrier consists of a monolayer of epithelium and tight junctions that seal the paracellular pathway and regulate permeability. Disrupting either of these two components can lead to a weakened barrier function manifested as “leaky gut”. Claudins are a family of proteins that are important in the formation of tight junctions (38). Among more than 10 Claudin family proteins, Claudin-2 is unique in that it is a pore-forming protein with the potential of increasing intestinal barrier permeability. Enhanced expression of Claudin-2 has been found to be associated with intestinal inflammatory disorders (35, 39). In our culture system, we found that TNF-α and IL-6 enhanced Claudin-2 expression in Caco-2 cells. Interestingly, RA appears to synergize with TNF-α and IL-6 to further enhance Claudin-2 expression. Thus, RA has the potential of increasing permeability of the epithelial monolayer in the presence of inflammatory cytokines. These results are different from other studies revealing a positive effect of RA on Caco-2 cells (40). The differences in experimental protocols, including the concentration of RA as well as the timing and duration of RA exposure may explain these different results. In addition, the interplay between RA and inflammatory cytokine signaling may dictate the positive or negative impacts of RA on the permeability of Caco-2 cells.

Exogenous RA has been shown to accelerate GVHD progression in animal models (18–20). Its detrimental effect has largely been attributed to increased Th1 cell differentiation and intestinal migration or decreased myeloid-derived suppressor cells. We found that exogenous RA can also enhance intestinal barrier permeability early after HSCT, as shown by significantly increased FITC-dextran levels in RA treated mice. Thus, RA may increase GVHD severity by acting on host nonimmune cells such as intestinal epithelial cells. This observation is noteworthy given the well-documented beneficial effects of vitamin A/RA in maintaining mucosal barrier integrity. It is worth noting that the complex effects of RA *in vivo* are often context dependent. For example, under steady state condition, RA acts as an anti-inflammatory molecule that dampens harmful immune responses in the GI tract by converting antigen-specific T cells into regulatory T cells (Tregs). However, in the presence of other inflammatory stimuli, such as IL-15, RA can act as an adjuvant that fuels T-cell mediated immune responses in this tissue site (41). By the same token, it is possible that RA promotes intestinal barrier integrity under homeostatic condition, but it can do the opposite in a highly inflammatory environment like the one observed in GVHD. An alternative explanation is that supplementation of exogenous RA induces a negative feedback regulation of RA production in intestinal epithelial cells, resulting in a low RA signaling in local tissue environment resembling vitamin A deficiency. It has been shown that RA actively induces the activity of *CYP26A1* and *CYP26B1* genes, which are responsible for RA breakdown *in vivo (* 42).

Vitamin A has been implicated in GVHD pathogenesis (36, 43–45). However, clinical studies have yielded some inconsistent conclusions. It has been reported that lower levels of vitamin A are associated with a significantly increased intestinal GVHD risk in pediatric patients undergoing allogeneic HSCT (46). In another study, however, pre-transplant serum vitamin A levels do not appear to affect the development of acute GVHD (47). The discrepancy between the two studies may originate from differences in study populations (pediatric *vs*. adult patients) and the timing of vitamin A measurement (post-transplant *vs*. pre-transplant). In addition, there is an interest in using vitamin A supplementation to reduce GVHD risk in the clinic (48). Therefore, it is imperative to test the applicability of this strategy in animal models of GVHD. In this study, we use a clinically relevant dose of vitamin A supplementation to examine how this approach influences the outcomes of allogeneic HSCT. It has been estimated that over one third of US population are taking dietary supplements regularly, including vitamin A (49). In addition, many types of foods are naturally fortified with vitamin A. Thus, there is a legitimate concern of relatively high prevalence of hypervitaminosis A in the population which may go unnoticed. Dietary vitamin A modification has been used extensively in various mouse models of human diseases. Many studies have used a vitamin A supplementation approach. In those experiments, a diet containing an excessive amount of vitamin A was given to experimental animals. However, most of those studies used aggressive vitamin A supplementation protocols that are not physiologically relevant; the vitamin A excessive diet is often 50-100 fold higher than normal levels. According to Food and Nutrition Board of Institute of Medicine, the Recommended Dietary Allowance (RDA) of vitamin A for men is 900 ug/day and the Tolerable Upper Intake Level (UL) is set at 3,000 ug/day, a roughly 3-fold difference (50). In our study, we used a 2.5-fold higher than normal vitamin A diet to mimic such a difference and ensured that our supplementation protocol is within UL and is clinically relevant. Consistent with the results from experiments using exogenous RA, VAH mice had increased serum LPS levels early after transplantation, indicating an increased epithelial barrier permeability. As discussed above, the additional strong inflammatory signals after allogeneic HSCT or a negative feedback mechanism with diminished local RA signaling in VAH animals may partially explain such a weakened mucosal barrier function. However, the increased gene expression of *aldh1a1* and *RAR-β* in VAH mice does not appear to support the latter possibility. Interestingly, gene expression of *Claudin-2* and apoptosis-associated molecules was unaltered in gut epithelial cells of VAH mice, suggesting that some other mechanisms may be responsible for the observation.

Donor T cells are the major pathogenic cells causing GVHD (51). We found no significant differences in donor T cell expansion and Treg reconstitution after BMT between VAN and VAH mice. Thus, vitamin A supplementation does not appear to directly affect the donor T cell compartment during GVHD. It is interesting that there was an increase in lung pathologic scores and transplant-associated mortality of VAH mice. It is generally accepted that vitamin A plays a beneficial role in maintaining lung homeostasis. However, it has also been shown that high dose vitamin A supplementation is associated an increased risk of lung cancer in humans, in particular among smokers (52). Thus, it appears that vitamin A could have negative impacts on lung health in the presence of inflammation. In addition, vitamin A levels may also affect the integrity of lung epithelium, thus influencing lung pathologic damage in our model.

Recent preclinical and clinical studies have shown that GVHD causes significant intestinal dysbiosis in recipients of allogeneic HSCT (53–60). There is also a strong interest in using probiotics, prebiotics, or fecal microbiota transplantation (FMT) to ameliorate GVHD severity by modifying gut microbiota (61–63). Diet is one of the most important factors that shape gut microbiota (64, 65). Micronutrients, including vitamin A, have been shown to modulate the gut microbiome community with functional consequences (66). In our study, we showed that a mere 2.5-fold difference in dietary vitamin A levels is sufficient to induce drastic differences in gut microbiota community in recipient mice after allogeneic BMT. VAH mice showed a higher degree of gut dysbiosis as demonstrated by significant changes in microbiota alpha and beta diversities post-BMT. They also had increased abundance of *Proteobacteria* and decreased abundance of *Firmicutes*, a characteristic dysbiosis frequently observed in patients after allogeneic HSCT or with inflammatory bowel disease (67–69). Furthermore, opportunistic pathogens such as *Escherichia-Shigella* were increased in VAH mice (70). On the other hand, *Bacteroides* and *Lactobacillus*, two bacterial genera that have the capacity to maintain intestinal homeostasis and health, were significantly lower in these animals. Thus, mild vitamin A supplementation, as defined in this study, leads to the expansion of pathogenic microbiota in the microbiome after transplantation. We further confirmed that changes in the diversity and composition of gut microbiota result in the alterations of bacterial metabolism. Functional pathway analysis revealed differences in amino acid, carbohydrate, and lipid metabolism between the two groups. Notably, *Bifidobacterium* shunt is among the top five pathways in VAN mice. *Bifidobacteria* are probiotics that can produce acetate, a type of short chain fatty acid, to promote gut health (71). In addition, pathway associated with lysine biosynthesis was increased in VAN mice, consistent with results from other studies (66). On the other hand, pathways associated with lipid and carbohydrate metabolism were increased in VAH mice. These data demonstrated that vitamin A supplementation can cause more profound gut dysbiosis with functional consequences in transplant recipients. The increased prevalence of pathogenic bacteria together with the alternations in energy homeostasis are also likely to contribute to the worse transplant outcomes of VAH mice. It is worth noting that changes in gut microbiota have been linked to the development of pulmonary complications in patients undergoing allogeneic HSCT (72). More studies focused on elucidating the role of micronutrients in regulating the interplay between intestinal epithelium, gut microbiota, and immune cells after allogeneic HSCT may lead to the development of novel strategies to prevent and/or treat GVHD.

There are some limitations to the current study. First, this study has not established a causal link between gut dysbiosis and increased lung pathology. Although there is clinical evidence supporting the notion that gut microbiota can influence the development of pulmonary complications in recipients of allogeneic HSCT (72), more studies in animal models are needed to provide mechanistic insights. Second, it is unclear whether similar gut dysbiosis will be observed in the absence of alloimmune response. While it is tempting to speculate that there will be less changes of gut microbiota in both VAN and VAH recipient mice in the absence of allogeneic T cells, bone marrow transplantations using TCD-BM alone will be needed to confirm this hypothesis. Third, we did not examine the microbial metabolites in this study. It is of interest to study whether alterations in gut microbiota diversity and composition lead to different levels of bacterial products such as short chain fatty acids (acetate, butyrate, and propionate etc) that have been shown to regulate GVHD severity.

**Figure 7:**
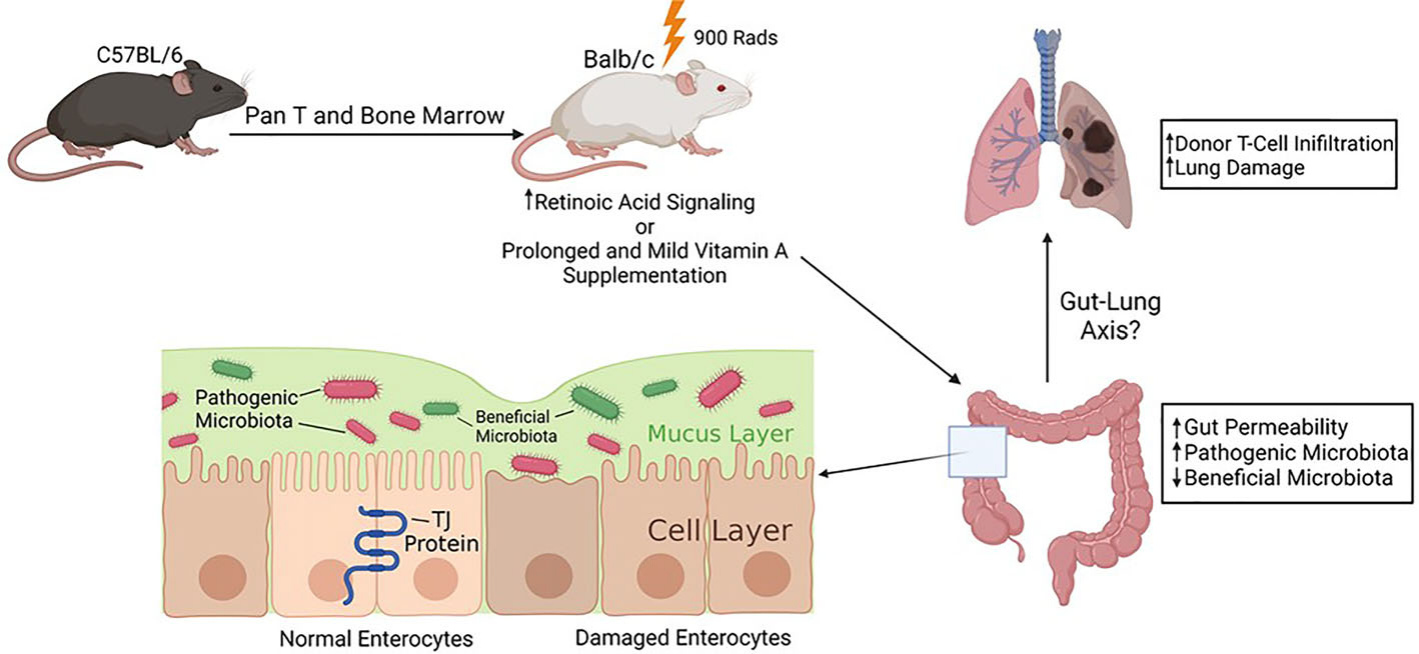
Schematic summary of the study. Increased retinoic acid signaling or prolong and mild vitamin A supplementation may negatively impact intestinal barrier function during GVHD. Mild vitamin A supplementation is associated with increased lung damage and more profound gut dysbiosis in recipient mice. Micronutrients such as vitamin A could modulate complications of allogeneic HSCT, which may be mediated by shaping gut microbiota. Created with Biorender.com.

In summary, our studies showed that RA signaling participates in modulating host intestinal epithelial barrier integrity and affects transplant outcomes (**Figure 7**). This effect may be mediated by the changes in gut microbiota in a vitamin A-dependent manner. In our model, it is possible that there is a reciprocal regulation between increased gut permeability and more profound gut dysbiosis in VAH recipient mice. The observation that VAH mice showed a significantly increased transplant-associated mortality in our model indicates that some caution should be exercised when consider using vitamin A supplementation to prevent GVHD in the clinic. However, we wanted to point out that we used a prolonged and very mild vitamin A supplementation protocol in this study. It is worth exploring whether a short-term, high dose vitamin A supplementation can produce a different result. Dietary intervention of BMT recipients aimed at correcting dysbiosis and improving intestinal epithelial barrier function may represent a clinically applicable, simple, and cost-effective approach for managing GVHD after allogeneic HSCT.

## Data Availability Statement

The datasets presented in this study can be found in online repositories. The names of the repository/repositories and accession number(s) can be found below: NCBI; PRJNA767073.

## Ethics Statement

All experiments and procedures were carried out under protocols approved by the MCW Institutional Animal Care and Use Committee.

## Author Contributions

PP performed research, analyzed data, and wrote the manuscript. SA performed all bioinformatic analyses of microbiota experiments. BT, HZ, DZ, and MM performed research. PF and LS-W discussed results. CL performed pathological analyses of GVHD target organs. IG performed hepatic function testing of experimental mice. XC conceived and supervised the study, designed experiments, analyzed data, and wrote the manuscript. All authors contributed to the article and approved the submitted version.

## Conflict of Interest

The authors declare that the research was conducted in the absence of any commercial or financial relationships that could be construed as a potential conflict of interest.

## Publisher’s Note

All claims expressed in this article are solely those of the authors and do not necessarily represent those of their affiliated organizations, or those of the publisher, the editors and the reviewers. Any product that may be evaluated in this article, or claim that may be made by its manufacturer, is not guaranteed or endorsed by the publisher.

## Abbreviations

ALT, alanine aminotransferase; AST, aspartate aminotransferase; BM, bone marrow; BMT, bone marrow transplantation; FMT, fecal microbiota transplantation; GVHD, graft-versus-host disease; GVL, graft-versus-leukemia; HSCT, hematopoietic stem cell transplantation; LDA, Linear discriminant analysis; LEfSe, Linear discriminant analysis effect size; LPS, lipopolysaccharide; MLNs, mesenteric lymph nodes; PCoA, Principal Coordinate Analysis; RA, retinoic acid; RDA, Recommended Dietary Allowance; Tregs, regulatory T cells; UL, Tolerable Upper Intake Level; VAH, vitamin A-high; VAN, vitamin A-normal.
